# Generation of homozygous canker‐resistant citrus in the T0 generation using CRISPR‐SpCas9p

**DOI:** 10.1111/pbi.13375

**Published:** 2020-04-01

**Authors:** Hongge Jia, Nian Wang

**Affiliations:** ^1^ Citrus Research and Education Center Department of Microbiology and Cell Science Institute of Food and Agricultural Sciences (IFAS) University of Florida Lake Alfred Fl USA

**Keywords:** susceptibility gene, citrus, CRISPR, *Xanthomonas*, *LATERAL ORGAN BOUNDARIES 1*, biallelic mutation, genome editing, disease resistance, Cas9, sgRNA

Citrus is the most produced fruit in the world and provides important nutrients such as vitamin C. However, citrus production worldwide faces many biotic and abiotic challenges. Citrus genetic improvement through traditional breeding is a lengthy, difficult and laborious process due to the long juvenility, nucellar embryony, sexual incompatibility, highly heterozygous nature, and male or female sterility. The clustered regularly interspaced short palindromic repeats (CRISPR)‐mediated genome editing has been suggested to be a putative solution for rapid improvement of existing citrus varieties (Dutt *et al.*, 2015).

Citrus canker caused by *Xanthomonas citri* subsp. *citri* (*Xcc*) is a severe bacterial disease in most citrus producing regions such as United States, Brazil and China. *Xcc* is known to cause canker symptoms via secretion of PthA4, a transcription activator like (TAL) effector, which activates the expression of the cognate susceptibility (*S*) gene *LATERAL ORGAN BOUNDARIES 1* (*LOB1*) gene via binding of effector binding elements (EBE) in the promoter region (Hu *et al.*, 2014; Li *et al.*, 2014). Consequently, researchers have aimed to generate canker‐resistant citrus varieties by mutation of the EBE or coding region of the *LOB1* gene using the CRISPR technology. However, no homozygous lines were generated in the previous work (Jia *et al.*, 2017; Peng *et al.*, 2017). Curiously, the S2 sgRNA (**3′**GAAAAGGAAAGAGATATATTTGG **5′**) targeting EBE_PthA4_‐CsLOBP used to generate biallelic lines (Peng *et al.*, 2017) is clearly inconsistent with the SpCas9 canonical sgRNA **5′** GN (19) NGG **3′** (Ma *et al.*, 2015). Here, we aimed to generate homozygous canker‐resistant citrus plants in the T0 generation using plant codon‐optimized SpCas9 (SpCas9p). Successful generation of homozygous citrus lines in the T0 generation is critical for timely citrus improvement due to the lengthy time needed to generate T1 plants.

Here, we used SpCas9p (Ma *et al.*, 2015) to modify the EBE region of the *LOB1* promoter in Pummelo (*Citrus maxima*). Pummelo is known for its large size and a popular fruit in Asia. It is a pure citrus species and is relatively homozygous (Wu *et al.*, 2018). We further confirmed the homozygous property of the promoter sequences of the *LOB1* gene in Pummelo via PCR and sequencing. Totally, 20 random colonies were sequenced and all of them contained one type of promoter sequence, Type II CsLOBP (Figure 1a; Jia *et al.*, 2016).

**Figure 1:**
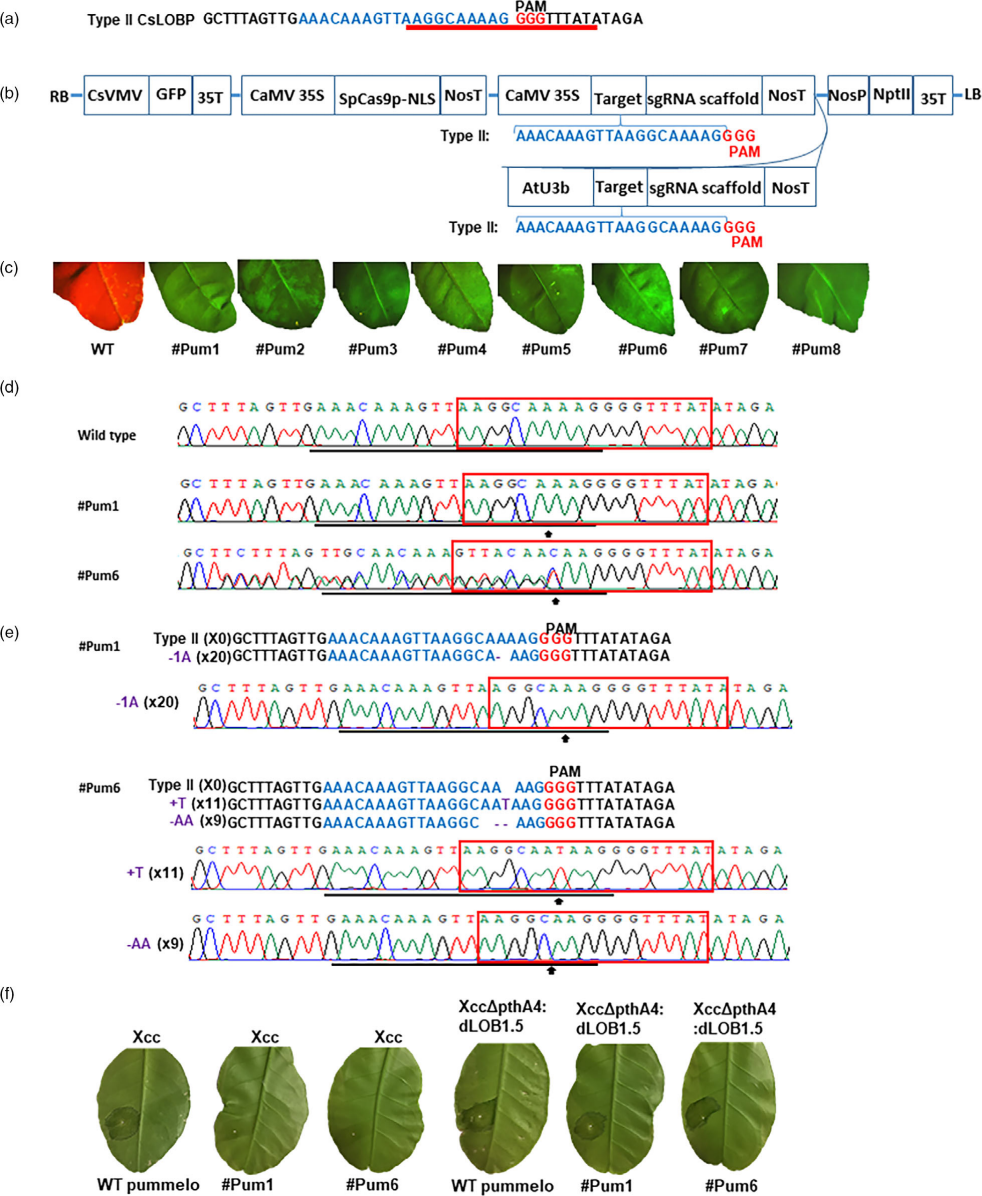
Generation of homozygous canker‐resistant citrus in the T0 generation using CRISPR‐SpCas9p. a. Type II CsLOBP in Pummelo. The PthA4 EBEs were highlighted by a red underline. A sgRNA was indicated in blue. b. Schematic diagram of GFP‐p1380N‐SpCas9p:PumLOBP. LB and RB, the left and right borders of the T‐DNA region; GFP, green fluorescent protein; CaMV 35S and 35T, the cauliflower mosaic virus 35S promoter and terminator; NosP and NosT, the nopaline synthase gene promoter and terminator; NLS, nuclear localization signal; AtU3b, *Arabidopsis* U3b promoter; NptII, neomycin phosphotransferase II. c. The GFP‐p1380N‐SpCas9p:PumLOBP‐transformed Pummelo were GFP positive. d. Sequencing analyses of GFP‐p1380N‐SpCas9p:PumLOBP‐transformed Pummelo. The mutant site or the beginning sites of double/multiple peaks were highlighted by arrows. The target was underlined and the EBE_pthA4_‐TII‐CsLOBP was highlighted by red rectangles. e. Chromatograms show the mutations in the homozygous line #Pum1 and biallelic line #Pum6. f. Five‐day post‐*Xcc* inoculation, canker symptoms were observed on wild‐type, but not on #Pum1 and #Pum6. Eight‐day post‐*Xcc*ΔpthA4:dLOB1.5 inoculation, canker symptoms were observed on wild‐type, #Pum1 and #Pum6.

We constructed a binary vector GFP‐p1380N‐SpCas9p:PumLOBP to edit EBE_PthA4_‐LOBP (Figure 1b). Since only the Type II CsLOBP is present in Pummelo, a single sgRNA was designed to target both alleles. To guarantee the efficient expression of sgRNA, we employed both AtU3b and CaMV35S to drive sgRNA expression (Figure 1b). The plant codon‐optimized SpCas9, designated as SpCas9p that was used to generate homozygous rice mutants in the first generation (Ma *et al.*, 2015), was adopted to develop binary vector GFP‐p1380N‐SpCas9p:PumLOBP (Figure 1b).

To test whether the resulting construct GFP‐p1380N‐SpCas9p:PumLOBP works in genome editing, *Xcc*‐facilitated agroinfiltration of Pummelo epicotyls was first carried out. Five days after agroinfiltration, genomic DNA was extracted from treated leaf tissue with the strongest GFP fluorescence. After PCR amplification, vector ligation and *Escherichia coli* transformation, 100 colonies were randomly selected for sequencing. Among them, two colonies contained the targeted indels in the LOBP sequence, indicating that GFP‐p1380N‐SpCas9p:PumLOBP is functional for modification of EBE_PthA4_‐LOBP.

Next, we conduct genome editing of Pummelo via *Agrobacterium*‐mediated transformation of epicotyls (Jia *et al.*, 2017). In total, eight transgenic lines, designated as #Pum1 to #Pum8, were successfully generated. The transgenic Pummelo plants were verified by PCR analysis and GFP observation (Figure 1c). Direct sequencing of PCR products was carried out to analyse mutations in the EBE_PthA4_‐LOBP among transgenic plants. Among them, #Pum1 was a homozygous mutant, since single peaks were present in its chromatogram with one adenine deleted from EBE_PthA4_‐LOBP (Figure 1d). #Pum6 was a biallelic mutant, since the dual sequencing signals were present in its chromatogram (Figure 1d). To verify the genotypes of #Pum1 and #Pum6, three different leaves from each line were further analysed with direct sequencing. The sequencing results indicated that their unique chromatograms were same as one another for #Pum1 or #Pum6. Thus, the results confirmed that #Pum1 was homozygous and #Pum6 was biallelic.

Subsequently, transgenic Pummelo plants were subjected to colony sequencing. Twenty random colonies were selected from each line. All Pummelo transformants contained the targeted EBE_PthA4_‐LOBP indels, with the mutation rates being 100%, 10%, 60%, 50%, 55%, 100%, 45% and 50% for #Pum1 to 8, respectively. Notably, both #Pum1 and #Pum6 had 100% mutation rates. It should be noted that #Pum1 contained only one type of indels, an adenine deletion within EBE_PthA4_‐LOBP (Figure 1d&e), whereas there were two types of indels in #Pum6, a thymine insertion within EBE_PthA4_‐LOBP in eleven colonies and two‐adenine deletion within EBE_PthA4_‐LOBP in nine colonies (Figure 1d&e). The results further confirmed that #Pum1 was a homozygous mutant and #Pum6 was a biallelic mutant.

We then tested the canker resistance of the EBE_PthA4_‐LOBP‐modified Pummelo lines and wild‐type Pummelo by inoculating with *Xcc* at the concentration of 5 × 10^8^ CFU/mL. At five days post‐inoculation (DPI), canker symptoms were observed on all of Pummelo plants except #Pum1 and #Pum6 lines (Figure 1f). The results indicated that the homozygous #Pum1 and the biallelic #Pum6 mutant lines in the EBE_PthA4_‐LOBP resist against *Xcc* infection.

To verify that the canker resistance of #Pum1 and #Pum6 was owing to EBE_PthA4_‐LOBP modification, wild‐type Pummelo and genome‐modified plants were inoculated with *Xcc*ΔpthA4:dLOB1.5. The dLOB1.5 was constructed to recognize the sequence 5′ TAAAGCAGCTCCTCCTCATCCCTT 3′, a sequence located downstream of the EBE but upstream of the ATG, that can activate the expression of the *LOB1* gene. Colony sequencing results indicated that dLOB1.5 binding sites were same in genome‐modified Pummelo plants as in the wild‐type Pummelo. As expected, *Xcc*ΔpthA4:dLOB1.5 caused typical canker symptoms on both wild‐type Pummelo and genome‐modified plants including #Pum1 and #Pum6 (Figure 1f).

Potential off‐target mutagenesis generated by SpCas9p:PumLOBP was analysed in the homozygous Pummelo line, #Pum1. By using a web tool (http://www.rgenome.net/cas‐offinder/), six potential off‐targets (mismatch number = 3) were present. However, it must be kept in mind that the reference sequence for off‐target finding is from sweet orange (http://www.rgenome.net/cas‐offinder/) that is a hybrid between Pummelo and mandarin (*C. reticulata*). It is very likely that there are single nucleotide polymorphisms between Pummelo sequence and sweet orange sequence. Overall, the colony sequencing results indicated that there were no off‐target mutations in the homozygous line #Pum1.

Generation of homozygous lines is required for crop breeding. Our results indicated that SpCas9p could be used to generate homozygous citrus lines in the first generation. Generating homozygous lines via the CRISPR technology has been completed for many crops including rice, maize and apple. Generation of homozygous mutations in the T0 plants via the CRISPR technology allows timely assessment of the targeted phenotypes and rapid improvement of elite citrus varieties. Interestingly, the efficacy to generate homozygous mutations in the T0 plants is variable. For example, its efficacy is very high for rice and maize, but is relatively low for wheat. For annual plants such as wheat, even low efficacy of generation of homozygous lines in the T0 generation is manageable since self‐pollination of T0 heterozygous plants allows easy development of homozygous lines. However, this is not the case for perennial tree crops, especially citrus considering its many challenges such as long juvenility. In citrus epicotyl transformation, most of the cells competent for transformation are the actively dividing cells located in the cambial ring of explants (Peña *et al.*, 2004). It appears that mutations in the homozygous and biallelic lines generated here happen before the first cell division of transformed cells in the epicotyl that eventually regenerated into whole plants via organogenesis.

In summary, our work demonstrates the generation of homozygous and biallelic canker‐resistant citrus plants in the T0 generation, representing an important breakthrough in the application of the CRISPR technology in citrus improvement. This study also provides a tool to rapidly identify genes whose mutations leading to resistance to Huanglongbing, the most devastating citrus disease worldwide (Wang *et al.*, 2017).

## Conflict of Interest Statement

The authors declare no conflict of interest.

## Author contributions

NW and HGJ designed and organized the study. HGJ conducted the experiments. NW and HGJ prepared the manuscript.
